# The Bidirectional Relationships Between Online Victimization and Psychosocial Problems in Adolescents: A Comparison with Real-Life Victimization

**DOI:** 10.1007/s10964-013-0003-9

**Published:** 2013-08-27

**Authors:** Regina van den Eijnden, Ad Vermulst, Antonius J. van Rooij, Ron Scholte, Dike van de Mheen

**Affiliations:** 1 Department of Interdisciplinary Social Sciences, Utrecht University, P.O. Box 80140, 3508 TC Utrecht, The Netherlands; 2 Behavioural Science Institute, Radboud University, Montessorilaan 3, 6525 HE Nijmegen, The Netherlands; 3 IVO Addiction Research Institute, Heemraadssingel 194, 3021 DM Rotterdam, The Netherlands; 4 Erasmus Medical Center Rotterdam, P.O Box 2040, 3000 CA Rotterdam, The Netherlands

**Keywords:** Internet, Victimization, Psychosocial factors, Internalization, Adolescent psychology, Longitudinal studies

## Abstract

Although peer victimization is of major concern and adolescents spend increasing amounts of time on the Internet, relatively little is known about the psychosocial antecedents and consequences of online victimization. The main aim of this study was to compare the psychosocial antecedents and consequences of online versus real-life victimization. More specifically, the bidirectional relationship between online and real-life victimization on the one hand and psychosocial problems (i.e., loneliness and social anxiety) on the other was examined. In addition, the moderating role of online aggression in the relationship between online victimization and subsequent psychosocial problems was studied. This prospective study, consisting of three annual measurements, was conducted among a sample of 831 adolescents (50.3 % girls) aged 11–15, of which most (80.2 %) had a Dutch ethnic background. The results indicate a unidirectional relationship whereby loneliness and social anxiety predict an increase in latter online victimization rather than the reverse. A bidirectional relationship was found for real-life victimization: loneliness (but not social anxiety) predicted an increase in latter real-life victimization, which in turn predicted an increase in subsequent social anxiety (but not loneliness). No moderating effects of online aggression were found. The findings of the present study suggest that negative online and in real life peer interactions have a differential meaning for, and impact on adolescents’ well-being.

## Introduction

It generally is believed that peer victimization is related negatively to the psychosocial well-being of adolescents. In agreement with this notion, positive associations are found between peer victimization, defined in terms of physical and verbal assaults (e.g., being hit, kicked or yelled at) as well as relational aggression (e.g., rumors being spread, not being allowed to take part in the group), and psychosocial problems such as loneliness, social anxiety, depression and low self-esteem [see e.g., the meta-analyses by Hawker and Boulton (2000)]. Theorists believe that peer victimization may cause psychosocial problems because victimization might be interpreted as negative peer evaluation or social exclusion. This may, in turn, reinforce negative self-evaluations (Lopez and DuBois 2005). Such self-evaluations may increase social anxiety and avoidance of social interactions (Crick and Bigbee 1998; Storch et al. 2005), thereby mounting feelings of loneliness and depression. Other theorists, however, emphasize that psychosocial problems also may be a precursor of peer victimization (Finnegan et al. 1996; Reijntjes et al. 2010). Adolescents who suffer from psychosocial problems such as social anxiety and loneliness generally will have more difficulties defending themselves effectively against online victimization (e.g., they often lack the skills and/or courage to defend themselves in response to peer victimization), and peers may take notice of this when they choose their victims for bullying. The association between peer victimization and psychosocial problems may thus result from two reverse underlying mechanisms. The goal of the present study was to examine to what extent these two mechanisms underlie the relationship between psychosocial problems and two forms of peer victimization, namely online and real-life victimization. More specifically, the goal of this study is to test the reciprocal relationship between online and real-life victimization on the one hand and adolescents’ psychosocial problems on the other.

Several longitudinal studies have examined unidirectional associations between psychosocial problems and peer victimization. From the studies that examined whether peer victimization predicted increases in psychosocial problems, most yielded positive findings (e.g., Arseneault et al. 2008; Bond et al. 2001; Goodman et al. 2001; Schwartz et al. 2005) but some did not (Khatri et al. 2000; Schwartz et al. 1998). Moreover, studies examining whether psychosocial maladjustment predicted increases in peer victimization showed inconsistent results (see for an overview Reijntjes et al. 2010). However, on the basis of their meta-analysis, which included 18 longitudinal studies, Reijntjes and colleagues concluded that there is a reciprocal relationship between psychosocial problems and peer victimization. These authors suggest a vicious circle whereby psychosocial problems increase the risk of peer victimization, and vice versa, peer victimization enhances psychosocial problems.

### Online Victimization

Most research addressing the link between peer victimization and psychosocial problems, including the meta-analysis by Reijntjes et al. (2010), has focused on victimization in real-life, mostly taking place in school classes or schools. Despite the central role of the Internet in adolescents’ lives, there is relatively little research on antecedents and consequences of online victimization. Although several cross-sectional studies have reported associations between online victimization and psychosocial problems in adolescents (Mitchell et al. 2007; Perren et al. 2010; Ybarra 2004; Ybarra et al. 2006), as far as we know, only two studies address the longitudinal change in psychosocial well-being following online victimization. The results indicate that online victimization is predictive of an increase in later emotional and depressive symptoms (Zwierzynska et al. 2013) and a decrease in life satisfaction later in life (Sumter et al. 2012). These studies, however, did not test the opposite longitudinal association whereby psychosocial well-being may predict changes in online victimization. Research examining the bidirectional association between online victimization and aspects of psychosocial well-being is thus scarce.

With regard to online victimization, it is important to emphasize that conclusions from research on peer victimization in real-life situations such as classes or schools cannot automatically be transferred to online victimization, since these two phenomena differ in many ways (Kiriakidis and Kavoura 2010; Slonje and Smith 2008; Tokunaga 2010). With regard to the effect of peer victimization on adolescents’ psychosocial problems, one noteworthy distinction is the high accessibility of the target through the Internet. Whereas real-life bullying mainly occurs during school hours and ceases once victims return home, bullying through e-mails, instant messengers and social network sites (e.g., Facebook) can take place at any given time of the day. It has been suggested that this aspect of online victimization may result in stronger negative psychosocial effects compared to real-life victimization (Tokunaga 2010). Another aspect that may augment the negative impact of online victimization is the fact that evidence of online harassments can reach a large audience, whereas real-life victimization is generally observed by a relatively small group. For instance, an embarrassing comment on one’s network site can become visible to a large peer audience. It also can be argued that online harassments may have longer-lasting negative effects because evidence of them may remain visible for a longer time, both to the victim as well as the audience. On the other hand, with regard to the opposite bidirectional association whereby psychosocial problems are assumed to predict subsequent peer victimization, it can be hypothesized that this association will be stronger for real-life than for online victimization, since psychosocial problems may be more noticeable and therefore more influential on peer behaviors in real-life settings than on the Internet.

### Online Versus Real-Life Victimization

When studying the reciprocal relationship between online victimization and psychosocial problems, it is crucially important to examine concurrently the psychosocial antecedents and consequences of real-life victimization, since previous studies have demonstrated that these two forms of victimization are correlated phenomena (Dehue et al. 2008; Erdur-Baker 2010; Juvonen and Gross 2008; Riebel et al. 2009) and are both related to psychosocial problems (Reijntjes et al. 2010; Sumter et al. 2012). Consequently, previous studies that focus exclusively on the psychosocial correlates of either online or real-life victimization are by definition unconvincing about the strength of these associations because found links also can be attributed to a third factor, namely victimization in the non-studied context (confounder effects). The aim of this study, therefore, was to compare the psychosocial antecedents and consequences of online versus real-life victimization, whereby two victimization related psychosocial problems were addressed, namely social anxiety (e.g., Crick and Bigbee 1998; Dempsey et al. 2009; Juvonen and Gross 2008; Storch et al. 2005) and loneliness (e.g., Catterson and Hunter 2010; Graham and Juvonen 1998; Hawker and Boulton 2000). These psychosocial problems are particularly interesting in relationship to experiencing victimization because they, more than for instance depression, seem to result from subjective appraisals regarding one’s functioning in peer and friend contexts.

As far as we know, no longitudinal data are available on the relative contribution of psychosocial problems to predicting online versus real-life victimization. On the one hand, it can be hypothesized that the predictive value of psychosocial problems on peer victimization will be stronger when occurring in a real-life than in an online setting, because psychosocial maladjustments will be more noticeable in a real-life context than on the Internet, and therefore more influential on peer aggressive behaviors. On the other hand, previous studies indicate that adolescents who experience psychosocial problems such as loneliness and social anxiety use the Internet to compensate for a lack of satisfying relationships in their real-life (Valkenburg and Peter 2007). As a result, they engage in online contact with “strangers”, i.e., people they meet on the Internet, more often (Campbell et al. 2006; Gross et al. 2002; Valkenburg and Peter 2007). Moreover, the risk of online victimization would be particularly high in case of online contact with strangers (Slovak and Singer 2011). As suggested by Valkenburg and Peter (2009), the anonymity of the source stimulates disinhibition of behavior and the lack of audiovisual information diminishes confrontation of the bully with the immediate effect of his or her act. Hence, because of their frequent online contact with strangers, adolescents with psychosocial problems can be assumed to have a higher risk of online victimization compared to the risk of real-life victimization.

### Online Aggression

Authors have suggested that it is important to distinguish between aggressive victims (those who combine aggression and victimization) and passive victims (victims only) (Craig 1998; Perry et al. 1988; Reijntjes et al. 2010). Previous studies indicate that passive victims of cyberbullying experience more psychosocial problems than aggressive victims (Kiriakidis and Kavoura 2010; Wang et al. 2011). Receiving a hostile message in response to one’s own offending behavior seems to be less upsetting and distressing than receiving such a message in a neutral social contact, probably because getting an intimidating message in reaction to one’s own aggression can be attributed to one’s own behavior, whereas getting such a message in a neutral context will be interpreted more often as evidence of social disapproval or rejection. The present study will therefore address the moderating role of online aggression on the relationship between online victimization and psychosocial problems, whereby it is assumed that passive victims (victims who *do not* engage in online aggression) will have a higher likelihood of developing psychosocial problems than active victims (victims who *do* engage in online aggression).

## Current Study

Empirical research on the reciprocal relationship between online victimization and psychosocial problems is scarce. It is unknown whether adolescents who are victimized online tend to develop psychosocial problems as a result (the “*effect hypothesis*”), or whether adolescents who experience psychosocial problems have a higher risk of being victimized online (the “*vulnerability hypothesis*”), or both. To gain more insight into the predominance of these two longitudinal associations, the present study will examine the bidirectional relationship between online victimization and adolescents’ psychosocial problems. In line with the literature on real-life victimization (see Reijntjes et al. 2010), we expect a reciprocal relationship whereby psychosocial problems predict subsequent online victimization and, vice versa, online victimization predicts future psychosocial problems (*Hypothesis 1*). These associations will be studied in a comprehensive model simultaneously testing the bidirectional relationship between adolescents’ psychosocial problems and real-life victimization. As far as we are aware, no insight exists into the relative severity of the psychosocial consequences of online versus real-life victimization. On the basis of the previously described higher accessibility of victims through the Internet and the fact that proof of online harassment will often reach a larger audience, in agreement with Tokunaga (2010) we hypothesized that the negative impact of online victimization will exceed that of real-life victimization (*Hypothesis 2*). In addition, because of their frequent online contact with strangers, adolescents with psychosocial problems can be assumed to have a higher risk of online victimization compared to the risk of real-life victimization. Therefore, it is hypothesized that psychosocial problems are more predictive of online than of real-life victimization (*Hypothesis 3*). Finally, the present study will address the moderating role of online aggression on the relationship between online victimization and psychosocial problems. More specifically, the hypothesis will be tested that passive victims (victims who *do not* engage in online aggression) will have a higher likelihood of developing psychosocial problems than active victims (victims who *do* engage in online aggression) (*Hypothesis 4*).

## Methods

### Procedure and Sample

This three-wave longitudinal study was conducted as part of an ongoing Monitoring Study “Internet and Youth” carried out by the IVO Addiction Research Institute. This study started in February/March 2006, and had annual follow-up measurements. Prior to data collection, all school boards granted permission and named a contact person who would be responsible for the administration of written questionnaires at the school. During the annual measurements, teachers were instructed by the contact person, and received precise written instructions about the classroom procedure, including guidelines to guarantee participants’ privacy while filling out the questionnaire.

Prior to participation, both parents’ and students’ passive informed consent was gathered. Parents received a letter in which they were informed about the fact that their child’s school was participating in a study on Internet use and well-being. If parents did not agree with their child’s participation, they could contact either the school board or the researchers. Students were told about all aspects of the study such as confidentiality of participation, and that they were free to decline or to withdraw from participation at any time.

A total of six secondary-level schools participated in this cohort. All schools were selected on the basis of school level (vocational training vs. high school or pre-university training) and region (north, east, south, and west), and were located in large- or medium-sized cities in the Netherlands. At the first measurement, all students in the 9th and 10th grades were selected for participation. During the follow-up measurements, students in the 10th and 11th grades (T2) and students in the 11th and 12th grades (T3) were asked to participate. At the first measurement, data were gathered among 1,777 students aged 11–15. Of this sample, 1,195 students also participated in the second measurement (response 67 %). A total of 836 students participated in all three measurements (response 47 %).

Attrition rates were mainly due to the fact that entire classes dropped out and not to individual student dropout. Attrition mainly resulted from two factors namely: (1) that our contact persons at the schools did not instruct all teachers, and (2) that schools did not want those classes to participate that were preparing for their final exam. The later was mainly the case in schools for vocational training. In addition, a number of students were missed because they had to repeat the previous year, had left school, or were absent on the day of measurement. Moreover, if we look at the within class participation rates, as one can expect with full-class participation, average per class participation is quite high namely around 90 %.

An attrition analysis was conducted to test whether respondents in the final sample (n = 836) differed from dropouts (n = 941). Some differences were identified in demographic variables: the final sample showed to be somewhat younger (OR .45, *p* < .001, 95 % .38 and .52), had a higher educational level (OR 1.28, *p* < .001, 95 % CI 1.11 and 1.48), and had a Dutch ethnic background more often (OR .63, *p* < .001, 95 % CI .50 and .79) than the dropouts (Nagelkerke R^2^ = .07). No differences were found for gender and for the relevant variables in this study (social anxiety, loneliness, online and real-life victimization, online contact with strangers, and online aggression).

The three-wave longitudinal sample consisted of 415 boys (49.7 %) and 420 girls (one missing value). The Mean age was 13.2 years at T1 (SD = .65) and ranged between 11.5 and 15.4. Of the students, 30 % followed vocational training, 37 % was in high school, and 33 % was in pre-university training. Most students (80.2 %) had a Dutch ethnic background.

### Measures

#### Loneliness

Feelings of loneliness were assessed with the 10-item Loneliness Scale (Russell et al. 1980). This scale contains 5 positive and 5 negative items. Examples of items are “I am feeling alone”, “I do not have real friends,” and “There are people who really understand me”. Negative items were recoded before summing the 10 items into a scale. Cronbach’s alpha ranged between .83 at T1 and .87 at T3.

#### Social Anxiety

We assessed social anxiety by two subscales of the Social Anxiety Scale for Children-Revised (SASC-R) (La Greca and Stone 1993). These subscales, the SAD-G (4 items) and SAD-New (6 items), measured “general social avoidance and distress” (SAD-G) and “social avoidance and distress specific to new situations” (SAD-New). An example of a SAD-G item is: “I feel shy even with kids I know very well”. An example of an SAD-New item is: “I get nervous when I talk to new kids”. Answer categories ranged from (1), “almost never” to (5) “always”. The original SASC-R also consisted of a subscale measuring “Fear of Negative Evaluations from Peers” (FNE). Because this subscale contained items conceptually similar to the online victimization scale (e.g., “I worry about being teased”, and “I feel that kids are making fun of me”), we did not use it. Cronbach’s alpha of the 10-item scale ranged from .86 at T1 to .89 at T3.

#### Online Victimization

Online victimization was assessed with a newly developed scale consisting of seven items. Adolescents were asked to give an indication of the frequency of online victimization in the last month. Questions asked: “How often have you been (1) bullied, (2) insulted, (3) treated rudely, (4) bothered, (5) ridiculed, (6) ignored, and (7) offended online”. Answers could be given on a 5-point scale ranging from: (1) “never”, (2) “about once a month”, (3) “about 2–3 times a month”, (4) “about once a week”, to (5) “more than once a week”. Cronbach’s alpha ranged between .83 at T1 and .89 at T3.

#### Real-Life Victimization

The scale to measure victimization in real-life consisted of the same seven items as were used to measure online victimization. Adolescents, however, were asked to give an indication of the frequency of these experiences in real-life. Cronbach’s alpha was .87 at T1 and T2, and .88 at T3.

#### Online Aggression

Online aggression also was measured with the same seven items as were used in the online victimization scale, but now from the position of the aggressor. Adolescents were asked to indicate how often they engaged in these behaviors in the last month. Cronbach’s alpha ranged from .77 at T1 to .89 at T3.

### Strategy of Statistical Analyses

First, correlations between the variables of interest are described. Next, structural equation modeling (SEM) was used to analyze 1-year follow-up longitudinal associations from T1 to T2 and from T2 to T3. The models were tested with MPLUS version 6.11 (Muthén and Muthén 1998–2010), using the Full Information Maximum Likelihood (FIML) estimator to deal with missing values. The percentage of missing values in this longitudinal sample varied between 0 and 3.2 %.

**Fig. 1:**
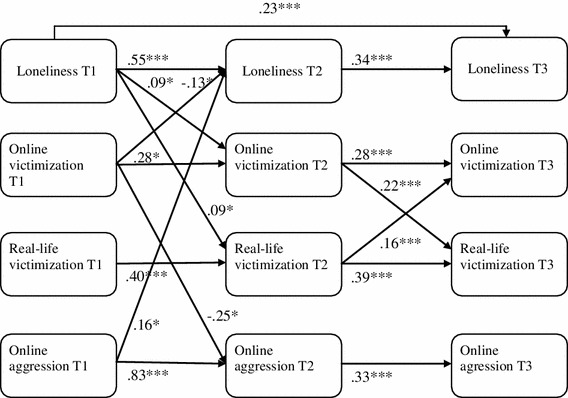
Cross-lagged model for loneliness, online victimization, real-life victimization and online aggression. *p < .05; ***p < .001

The variables loneliness, social anxiety, online victimization, real-life victimization and online aggression are treated as latent variables. Because the number of parameters to be estimated in the model of Fig. 1 would increase rapidly by using items as indicators for the latent variables with the consequence that power to detect important parameters will decrease (Yang et al. 2010) and estimation problems will increase (Sass and Smith 2006), we decided to use three parcels as indicators for each of the latent variables. The items of each concept were split up into three equivalent parts (parcels). First, a one-factor solution was implemented with all items belonging to a latent concept at T1. Next, items were allocated to parcels according to the magnitude of the factor loadings, with each parcel containing items with higher and lower factor loadings. By using this method, which is called item-to-construct balance (Little et al. 2002), the parcels optimally reflected the original one-factor structure. The items of the parcels at T1 were identical to those of T2 and T3.

Two fit measures were used, as recommended by several authors: (1) the Root Mean Square Error of Approximation (RMSEA) (Byrne 1998; Kaplan 2000) and (2) the Comparative Fit Index (CFI) of Bentler (Kaplan 2000; Kline 1998). RMSEA values lower than or equal to .05 are preferred, but under .08 are acceptable, and CFI values above .95 (.90) are indicative of a fair (acceptable) fit. To be sure that the standard errors of the parameter estimates are corrected for possible skewness of the variables we used the Robust Maximum Likelihood (MLR) estimator. Because students were nested within schools, we corrected for possible nonindependence of the data by applying the COMPLEX procedure in Mplus to get unbiased estimates of the standard errors of the parameters.

**Fig. 2:**
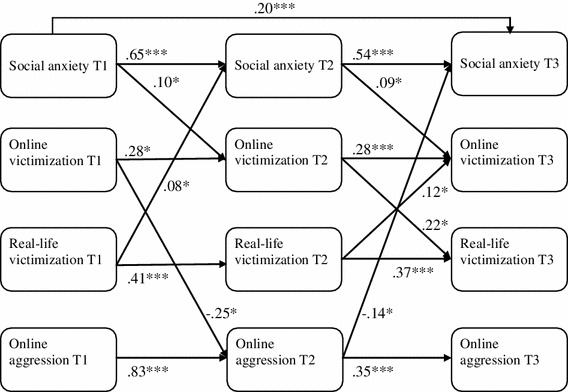
Cross-lagged model for social anxiety, online victimization, real-life victimization and online aggression. *p < .05; ***p < .001

Prior to the final analyses, we tested the measurement part of the latent variables in the models of Figs 1 and 2. Loneliness, social anxiety, online victimization, real-life victimization and online aggression at T1, T2 and T3 were put in Confirmatory Factor Analysis (CFA) with parcels as indicators of the latent variables. Error terms of corresponding parcels over time were allowed to correlate as recommended by Finkel (1995) for longitudinal models. The fit of the factor model was χ^2^(795) = 1,400.65, *p* < .001, CFI = .973 and RMSEA = .030, indicating a good model fit. The standardized factor loadings were substantial and varied between .68 and .97 (M = .84, SD = .06). The standardized factor loadings of the CFA are reported in Table 1 and are (almost) identical with the factor loadings of the measurement parts of the cross-lagged models described below.

**Table 1:** Factor loadings of the five concepts of Figs. 1 and 2

	T1			T2			T3		
	Parcel 1	Parcel 2	Parcel 3	Parcel 1	Parcel 2	Parcel 3	Parcel 1	Parcel 2	Parcel 3
Loneliness	.68	.76	.86	.77	.73	.91	.76	.79	.97
Social anxiety	.86	.82	.86	.88	.85	.83	.90	.86	.89
Online victimization	.83	.74	.85	.90	.82	.88	.91	.86	.93
Real-life victimization	.85	.78	.86	.84	.79	.90	.88	.81	.90
Online aggression	.77	.71	.80	.84	.80	.85	.90	.88	.89

Two cross-lagged panel analyses were conducted to test the longitudinal reciprocal cross-lagged associations: the first model with loneliness, online victimization, real-life victimization and online aggression (Fig. 1) and the second model with loneliness replaced by social anxiety (Fig. 2). The first aim of the analysis was to test the bidirectional relationship between online victimization and psychosocial problems (i.e., loneliness and social anxiety), and to determine whether one of the cross-relations is predominant (Finkel 1995) while controlling for two factors that are related to online victimization namely real-life victimization (Dehue et al. 2008; Erdur-Baker 2010; Juvonen and Gross 2008; Riebel et al. 2009) and online aggression (Mishna et al. 2012; Wang et al. 2011). The second aim was to test the relative impact of online versus real-life victimization on psychosocial problems of adolescents. These cross-lagged analyses were performed with all latent variables of the model regressed on the control variables gender, age, ethnic background and educational level.

To test the moderating role of online aggression on the relationship between online victimization and loneliness (social anxiety) from T1 to T2 and from T2 to T3, we determined latent interaction terms. For this test, the interaction online aggression T1 × online victimization T1 was included as predictor of loneliness T2 (social anxiety T2), and the interaction online aggression T2 × online victimization T2 was included as predictor of loneliness T3 (social anxiety T3). Interaction terms were highly non-normal and required numerical integration. In Mplus the Latent Moderated Structural equations (LMS) approach of Klein and Moosbrugger (2000) was used. Including the tests simultaneously was not possible due to limited memory space.

## Results

### Descriptive Statistics and Correlations

Descriptive statistics of the variables of interest are given in Table 2. Correlations between manifest variables (computed as the mean of the items of a concept) were computed for all three waves, see Table 3. Both within and across the three time points, loneliness and social anxiety were related significantly to online victimization (correlations varying between .11 and .20) and real-life victimization (correlations varying between .11 and .29), except for the association between social anxiety at T1 and online victimization at T3. These findings indicate that adolescents with higher levels of loneliness and social anxiety are at greater odds of being victimized on the Internet as well as in real life. High cross-sectional and longitudinal correlations also were found between online aggression and online victimization (correlations ranging between .34 and .64), suggesting that adolescents who engage in online aggression have higher chances of being or becoming online victims. Finally, moderate-to-high one- and 2-year stability correlations were found for loneliness (between .39 and .51), social anxiety (between .49 and .62), online aggression (between .37 and .52), online victimization (between .35 and .42), and real-life victimization (between .32 and .46).

**Table 2:** Mean, standard deviation and skewness for the variables of interest

	T1			T2			T3		
	M	SD	Skewness	M	SD	Skewness	M	SD	Skewness
1. Loneliness	1.60	.47	.84	1.57	.48.	1.04	1.60	.54	1.04
2. Social anxiety	2.07	.64	.64	2.05	.63	.49	2.06	.68	.49
3. Online victimization	1.43	.58	2.36	1.35	.56	2.83	1.33	.61	2.83
4. Online aggression	1.30	.46	2.87	1.26	.48	3.70	1.28	.56	3.70
5. Real-life victimization	1.47	.63	2.16	1.54	.64	2.26	1.51	.65	2.26

**Table 3:** Correlations between loneliness, social anxiety, online aggression, online victimization and real-life victimization across three waves

Variables	1.	2.	3.	4.	5.	6.	7.	8.	9.	10.	11.	12.	13.	14.
Wave 1														
1. Loneliness	–													
2. Social anxiety	.**43**	–												
3. Online aggression	−**.09**	.07	–											
4. Online victimization	**.18**	**.13**	**.57**	–										
5. Real-life victimization	**.26**	**.20**	**.39**	**.50**	–									
Wave 2														
6. Loneliness	**.51**	**.33**	**.12**	**.11**	**.18**	–								
7. Social anxiety	.**35**	**.62**	−.02	.06	**.17**	**.49**	–							
8. Online aggression	.02	−.01	**.52**	**.25**	**.13**	**.09**	−.01	–						
9. Online victimization	**.17**	**.14**	**.35**	**.42**	**.27**	**.19**	**.13**	**.64**	–					
10. Real-life victimization	**.21**	**.14**	**.29**	**.34**	**.46**	**.29**	**.19**	**.35**	**.50**	–				
Wave 3														
11. Loneliness	**.39**	**.22**	**.14**	**.09**	**.13**	**.48**	**.30**	.07	**.15**	**.17**	–			
12. Social anxiety	**.28**	**.49**	.01	.02	**.11**	**.35**	**.59**	−.06	.08	.09	**.46**	–		
13. Online aggression	**.08**	−.00	**.37**	**.27**	**.16**	.01	.01	.**42**	.**36**	**.24**	**.22**	.01	–	
14. Online victimization	**.13**	.07	**.34**	**.35**	**.23**	**.11**	**.12**	.**36**	**.44**	**.34**	**.20**	**.12**	**.60**	–
15. Real-life victimization	**.18**	**.11**	**.25**	**.30**	**.32**	**.16**	**.11**	**.32**	**.41**	**.48**	**.27**	**.18**	**.44**	**.54**

The bold figures are significant at *p* < .05; the bold and underscored figures are significant at *p* < .01 (2-tailed)

### The Bidirectionality Between Adolescents’ Psychosocial Problems and Online and Real-Life Victimization

Figure 1 shows the longitudinal cross-lagged model for loneliness, online victimization, real-life victimization and online aggression. The model showed good model fit (χ^2^ (605) = 1,262.04, *p* < .001, CFI = .9564 and RMSEA = .036). While controlling for autoregressive and concurrent associations, loneliness at T1 significantly predicted online victimization T2 (*β* = .09, *p* < 0.05) and real life victimization T2 (*β* = .09, *p* < 0.05). Also, an opposite pathways was found from online victimization T1 to subsequent loneliness at T2 (*β* = −.13, *p* < 0.05). Surprisingly, this association showed to be negative, indicating that online victimization at T1 would decrease feelings of loneliness at T2. In addition, online victimization at T1 negatively predicted online aggression at T2 (*β* = −.25, *p* < 0.05), online aggression at T1 predicted loneliness at T2 (*β* = .16, *p* < 0.05), online victimization at T2 predicted real-life victimization at T3 (*β* = .22, *p* < 0.05), and real-life victimization at T2 also predicted online victimization at T3 (*β* = .16, *p* < 0.01).

Figure 2 depicts the longitudinal cross-lagged model for social anxiety, online victimization, real-life victimization and online aggression. The model showed good model fit (χ^2^ (605) = 1,114.60, *p* < .001, CFI = .967 and RMSEA = .032). While controlling for autoregressive and concurrent associations, a significant pathway was found from social anxiety at T1 to online victimization at T2 (*β* = .10, *p* < .05) and from social anxiety at T2 to online victimization at T3 (*β* = .09, *p* < .05). The absence of significant cross paths from online victimization to social anxiety from T1 to T2 and from T2 to T3 indicates that social anxiety is a predominant predictor of online victimization (the more social anxiety, the more online victimization). Real-life victimization at T1 was a significant predictor of social anxiety at T2 (*β* = .08, *p* < .05) (the more real-life victimization, the more social anxiety), online victimization at T1 was a significant negative predictor of online aggression at T2 (*β* = −.25, *p* < .05) (the more online victimization, the less online aggression), and online aggression at T2 a significant predictor of social anxiety at T3 (*β* = –.14, *p* < .05) (the more online aggression, the less social anxiety). Finally, similarly to Fig. 1, two significant cross lagged relationships were found between online victimization and real-life victimization and between real-life victimization and online victimization from T2 to T3 (*β* = .22, *p* < 0.05 and *β* = .12, *p* < 0.05 respectively).

In sum, with regard to the hypothesis that a reciprocal relationship would exist between psychosocial problems and online victimization (Hypothesis 1), the present findings only yielded evidence that psychosocial problems (i.e., feelings of loneliness and social anxiety) would increase the risk of subsequent online victimization. There were no indications that online victimization would increase subsequent psychosocial problems. Instead, the results suggest that online victimization may protect against later feelings of loneliness. These findings are supported by additional analyses testing the difference between the path from loneliness at T1 to online victimization at T2 and the path from online victimization at T1 to loneliness at T2. The Chi square difference test showed a significant difference between the unconstrained model and the model with the two paths constrained to be equal: Δχ^2^(1) = 9.88, *p* < .01). In the same manner, we compared the path from social anxiety to online victimization with the path from online victimization to social anxiety from T1 to T2 and from T2 to T3. The Chi square difference test showed a significant difference between the unconstrained model and the constrained model with the cross-lagged paths constrained to be equal from T1 to T2 and from T2 to T3: Δχ^2^(2) = 8.41, *p* < .05). In both cases, the difference tests showed that the model fit of the unconstrained models were significantly better than those of the constrained model, leading to the conclusion that the cross paths from psychosocial problems to online victimization and vice versa are not equal.

Hypothesis 2, which predicted that the negative impact of online victimization would exceed the negative impact of real-life victimization, was not supported by the present data. Real-life victimization predicted an increase in one of the psychosocial problems, i.e., social anxiety, whereas online victimization did not. This result also is supported by analyses testing the difference between the path from online victimization at T1 to social anxiety at T2 and the path from real-life victimization at T1 to social anxiety at T2. The Chi square difference test showed a significant difference between the unconstrained model and the model with the two paths constrained to be equal: Δχ^2^(1) = 5.28, *p* < .05). The model fit of the unconstrained model was significantly better than that of the constrained model, indicating that the two paths are not significantly equal. Hypothesis 2 was neither supported by the findings for loneliness. Whereas real-life victimization did not significantly affect later loneliness, as described before, online victimization even predicted a decrease in later loneliness, suggesting that online victimization would prevent later feelings of loneliness.

Finally, the findings are in line with Hypothesis 3, which states that psychosocial problems are more predictive of online than of real-life victimization. While the loneliness model showed similarly strong associations with later online and later real-life victimization, the social anxiety model showed that social anxiety was predictive of online victimization but not of real-life victimization.

### Moderation by Online Aggression

To test hypothesis 4, that online aggression would moderate the relationship between online victimization and psychosocial problems, we inserted the latent interaction terms of online aggression × online victimization (at T1 and T2) to predict loneliness at T2 and T3. These interaction terms appeared to be non-significant with *B* = .03, *p* > .05 and *B* = −.01, *p* > .05 respectively. The same procedure was followed for social anxiety. The interaction terms online aggression × online victimization (at T1 and T2) predicting social anxiety at T2 and T3 did not show to be significant with *B* = .01, *p* > .05 and *B* = −.04, *p* > .05 respectively. Online aggression is not a significant moderator of the relationship between online victimization and psychosocial problems.

## Discussion

A recent meta-analysis on peer victimization in children and adolescents indicated a reciprocal relationship between psychosocial problems and online victimization (Reijntjes et al. 2010). This meta-analysis, however, exclusively focused on victimization in real-life situations. Since adolescents spend increasing amounts of time on the Internet, the aim of the present study was to test the reciprocal relationship between adolescents’ psychosocial problems (i.e., loneliness and social anxiety) and online victimization. A second aim was to test whether online victimization would have more negative consequences for adolescents’ psychosocial well-being than real-life victimization (cf. Tokunaga 2010), and whether having psychosocial problems would be more predictive of online than of real-life harassments. Because of the link between online and real-life victimization, these questions were addressed by testing a comprehensive longitudinal model concurrently examining the bidirectional relationships between adolescents’ psychosocial problems on the one hand, and online and real-life victimization on the other.

The findings of the current study suggest a unidirectional relationship between online victimization and psychosocial problems whereby feelings of loneliness and social anxiety predict an increase in later online victimization rather than the reverse. In line with Reijntjes et al. (2010), a bidirectional relationship was found between real-life victimization and psychosocial problems: loneliness (but not social anxiety) predicted an increase in latter real-life victimization, which in turn predicted an increase in subsequent social anxiety (but not loneliness). Accordingly, the present findings suggest that real-life victimization has more negative effects for the psychosocial well-being of adolescents than online victimization. The results, furthermore, indicate that psychosocial problems are more predictive of online than of real-life victimization. While loneliness predicted both later online and real-life victimization, social anxiety was only predictive of later online victimization (and not real-life victimization). Finally, no evidence was found for a moderating role of online aggression on the relationship between online victimization and later psychosocial problems in adolescents.

Although several authors implied that online victimization may impose an important risk for the psychosocial health of adolescents (Mitchell et al. 2007; Perren et al. 2010; Sumter et al. 2012; Ybarra 2004; Ybarra et al. 2006), the present findings do not support this *effect hypothesis* for online victimization, but are in line with the *vulnerability hypothesis* suggesting that socially vulnerable adolescents have a higher risk of being victimized online. As mentioned before, a possible explanation for this finding may be given by research showing that socially vulnerable adolescents seem to engage in online contact with strangers more often (Campbell et al. 2006; Gross et al. 2002; Valkenburg and Peter 2007), and that online communication with strangers is related positively to online victimization (Slovak and Singer 2011). Future research should focus on the mechanisms that cause lonely and socially anxious adolescents to be relatively more vulnerable to the experience of online victimization.

There is no evidence that online victimization would have more negative consequences because of the higher accessibility of victims through the Internet and the fact that proof of online harassment reaches a larger audience (Tokunaga 2010). Instead, even a positive effect was found whereby online victimization predicted a decrease in later loneliness. Since the latter finding may be an artifact of our multivariate analyses, replication of this finding is warranted before any conclusions can be drawn about the effect of online victimization on subsequent feelings of loneliness among adolescents.

The present study suggests differential associations between real-life victimization and the two psychosocial problems under study. Whereas loneliness predominantly seems to be a precursor of real-life victimization, social anxiety mainly seems to be the result of real-life harassments. A possible explanation for the result that loneliness predicts real-life victimization may be that loneliness, more than social anxiety, is related to social isolation. It seems that bullies choose their victims more or less deliberately (Finnegan et al. 1996; Reijntjes et al. 2010), whereby relatively isolated victims who do not have a social backup of peers will be most suitable for victimization because they will provide the lowest risk of social repercussions. Social anxiety, on the other hand, more than loneliness, may be a fear response to the experience of real-life victimization, underlining that peer victimization may interfere with important developmental processes and may boost emotional problems in adolescents (Prinstein et al. 2001; Zwierzynska et al. 2013).

The present study yielded some additional results. First, in line with previous studies (e.g., Campfield 2008), the present data indicate that the risk of online victimization increases as a result of real-life victimization, and vice versa, that the risk of real-life victimization increases as a consequence of online victimization. In this regard, it is important to note that there is large overlap between online and real-life networks. The online network of most Dutch adolescents is made-up of friends and peers known from the real world (Valkenburg and Peter 2007). It seems that victimization in one of the two social contexts raises the probability of being bullied in the other context, for instance because the victim will probably meet the perpetrator in both social contexts. Second, the findings indicate that online aggression may enhance feelings of loneliness, whereas it seems to prevent feelings of social anxiety. These findings are remarkable because there is hardly any evidence for an association between online aggression and internalizing problems (Campfield 2008; Juvonen et al. 2003; Nansel et al. 2004)—although the finding that online aggression increases feelings of loneliness is in line with a study by Wilson (2004), showing that youth who perpetrate violent behavior are less likely to feel connected to others at their school. Finally, a result that we did not anticipate was that the experience of online victimization seems to lower the chances of future online aggression. This finding is not in line with previous studies, indicating that victimization and aggression are positively associated constructs (e.g., Werner et al. 2010). Since the present study showed strong positive correlations between online victimization and online aggression, the negative impact of online victimization on subsequent online aggression may be an artifact of our multivariate analyses as well. Future longitudinal research with a similar design is warranted to determine with more certainty the impact of online aggression on adolescents’ feelings of loneliness and social anxiety, as well as the impact of online victimization on later online aggression.

The present study has some strong aspects. It used a longitudinal design including three annual measurements and used cross-lagged models to simultaneously test the bidirectional relationship between psychosocial problems and online victimization. In addition, the present study is unique for using a comprehensive model controlling for important confounders such as real-life victimization and online aggression while testing the bidirectional relationship between psychosocial problems and online victimization.

Some limitations of the present study have to be mentioned as well. First, the results are based on adolescents’ self-reports about psychosocial problems, online and real-life victimization, and online aggression. There is some research indicating that children with a negative cognitive bias will interpret ambiguous situations more easily as an incidence of peer victimization (Rosen et al. 2007). It can be assumed that lonely and socially anxious adolescents will have more negative cognitive schemes and therefore may have more subjective experiences of peer victimization, resulting in higher self-reports. A second limitation is that we used new scales to measure online and real-life victimization. Because already existing scales for online and real-life victimization showed important differences, these scales were not suitable for the aim of the present study to compare the strength of the longitudinal relationships between online and real-life victimization and psychosocial problems. We, therefore, had to develop two new identical scales. Although the items showed good factor loadings and the scales demonstrated satisfactory internal reliabilities, we do not have any information about the validity of the scales.

## Conclusions

The present findings provide convincing evidence that feelings of loneliness and social anxiety are risk factors for experiencing online victimization. In addition, there is some evidence that loneliness, but not social anxiety, is a risk factor for real-life victimization. On the basis of these differential results, it seems that socially vulnerable adolescents are somewhat more susceptible to experiencing online victimization than real-life victimization. On the other hand, the negative impact of real-life victimization seems to exceed the negative impact of online victimization. Although several arguments have been proposed to underpin that online victimization may be more harmful for the psychosocial well-being of adolescents than victimization in real-life, the opposite seems to be the case. Victimization in real-life seems to augment feelings of social anxiety in adolescents, whereas online victimization does not.

Even though the precise mechanisms that operate in the relationship between peer victimization (on the Internet and in real-life) and adolescents’ psychosocial problems are not fully understood, the present findings add to a growing body of literature indicating that peer victimization hinders a healthy psychosocial development by increasing internalizing problems in adolescents, and that a risk group of adolescents can be identified (e.g., Reijntjes et al. 2010). Peer victimization, therefore, should be considered an important public health risk, rather than a tolerable aspect of adolescent life (Srabstein 2009). The present study clearly shows that online and real-life victimization are mutually reinforcing phenomena whereby the risk of being victimized in one context (either online or in real-life) increases the risk of similar experiences in the other context. Therefore, it is vital that (future) prevention programs addressing peer victimization not only focus upon real-life victimization (e.g., Jiménez Barbero et al. 2012), but also aim to prevent online victimization. Furthermore, since a risk group for online victimization can be identified on the basis of already-existing psychosocial problems, public health researchers and workers in the field of peer victimization should consider whether selective preventive interventions aiming at high risk youth should become part of general (school-based) prevention programs.

It is widely acknowledged that adolescence is a period during which peer relationships become of crucial importance for adolescents’ development and well-being (e.g., Scholte and van Aken 2006). Most theoretical models equate peer relationships and interactions with “real life” relationships and interactions. The findings from our study reveal that the social arena of adolescents nowadays is much more complex and include both real life and online peer interactions, that have a differential meaning for, and impact on adolescents’ well-being. Finally, it seems crucially important to teach adolescents and parents about the risks of engaging in online contact with strangers and to provide them with tools on how to interpret and deal with these experiences.
